# A study protocol of qualitative data sharing practices in clinical trials in the UK and Ireland: towards the production of good practice guidance

**DOI:** 10.12688/hrbopenres.13269.1

**Published:** 2021-05-10

**Authors:** Catherine Houghton, Megan McCarthy, Katie Gillies, Nikki Rousseau, Julia Wade, Carrol Gamble, Elaine Toomey, Karen Matvienko-Sikar, Matthew Sydes, Maura Dowling, Val Bryant, Linda Biesty

**Affiliations:** 1 School of Nursing and Midwifery, National University of Ireland Galway, Galway, Ireland; 2 Health Services Research Unit, University of Aberdeen, Aberdeen, UK; 3 Leeds Institute of Clinical Trials Research, University of Leeds, Leeds, UK; 4 Bristol Population Health Science Institute, University of Bristol, Bristol, UK; 5 Health Data Science, University of Liverpool, Liverpool, UK; 6 School of Allied Health, University of Limerick, Limerick, Ireland; 7 School of Public Health, University College Cork, Cork, Ireland; 8 Clinical Trials Conduct Methodology, University College London, London, UK; 9 No particular affiliation, No particular affiliation, UK

**Keywords:** qualitative, data sharing, trials, focus groups

## Abstract

**Background**: Data sharing enables researchers to conduct novel research with previously collected data sets, thus maximising scientific findings and cost effectiveness, and reducing research waste. The value of sharing anonymised data from clinical trials is well recognised with a moderated access approach recommended. While substantial challenges to data sharing remain, there are additional challenges for qualitative data. Qualitative data including videos, interviews, and observations are often more readily identifiable than quantitative data. Existing guidance from UK Economic and Social Research Council applies to sharing qualitative data but does not address the additional challenges related to sharing qualitative data collected within trials, including the need to incorporate the necessary information and consent into already complex recruitment processes, with the additional sensitive nature of health-related data.

**Methods: **Work package 1 will involve separate focus group interviews with members of each stakeholder group: trial managers, clinical trialists, qualitative researchers, members of research funding bodies and trial participants who have been involved in qualitative research. Data will be analysed using thematic analysis and managed within QSR NVivo to enhance transparency. Work package 2 will involve a documentary analysis of current consent procedures for qualitative data collected as part of the conduct of clinical trials. We will include documents such as participant information leaflets and consent forms for the qualitative components in trials. We will extract data such as whether specific clauses for data sharing are included in the consent form. Content analysis will be used to analyse whether and how consent is being obtained for qualitative data sharing.

**Conclusions:** This study will provide insight into the existing practice of sharing of qualitative data in clinical trials and the current issues and opportunities, to help shape future research and development of guidance to encourage maximum learning to be gained from this valuable data.

## Introduction

Data sharing enables researchers to conduct novel research with previously-collected data sets, thus maximising scientific findings and cost effectiveness, and reducing research waste ( DuBois *et al.*, 2018). The value of sharing data in clinical trials is well recognised, with a moderated (controlled) access approach recommended ( Sydes *et al.*, 2015), and guidelines exist for how this should be done ( Keerie *et al.*, 2018; Institute of Medicine, 2015; Ohmann *et al.*, 2017). While substantial challenges to data sharing remain, there are additional challenges for qualitative data ( NASEM, 2020). This is particularly evident in the context of general data protection regulation (GDPR) in Europe and its implementation in the Data Protection Act (2018), where an individual’s right to the privacy of their personal data is paramount. There are specific challenges in sharing qualitative data, including videos, interviews, and observations, which are often more readily identifiable than quantitative data. Therefore, concerns for privacy become challenging, specifically regarding pseudonymisation which has been identified as a major barrier to data sharing ( Aitken *et al.*, 2016; Ruggiano & Perry, 2019). Pseudonymisation can be described a technique that replaces or removes information in a data set that identifies an individual ( Mourby *et al.*, 2018). Furthermore, while the public may assume or expect their quantitative data to be shared, they may not be clear or comfortable with sharing their qualitative data ( Aitken *et al.*, 2016).

It is well recognised that qualitative components of trials are valuable for: developing further research hypotheses; gathering complementary information to contribute to answering research questions in depth and helping to explain findings ( Rapport *et al.*, 2013). In addition, qualitative research in trials can be particularly helpful in developing and evaluating complex interventions as it provides valuable insights to issues experienced by potential participants ( Rapport *et al.*, 2013). However, there is little guidance on how to approach sharing this type of data. Guidance from UK economic and social research council applies to sharing qualitative data generally, but qualitative research in trials faces additional challenges, including the need to incorporate the necessary information and consent into already complex recruitment processes, with the additional sensitive nature of health-related data. Consequently, investigators lack proper guidance on how to comply with data sharing guidelines in a way that provides adequate anonymity protections ( Tsai *et al.*, 2016).

To this end, the aim of the project is to explore whether and how trial teams share qualitative data collected as part of the design, conduct or delivery of clinical trials. This project will provide the foundation for further methodological work and the future production of guidance by exploring potential challenges and opportunities when considering qualitative data sharing in trials.

## Methods

### Work package 1

***Study design.*** This study will employ a qualitative descriptive approach using thematic analysis of data ( Braun & Clarke, 2006). This approach explores general beliefs and views that expose the experiences described by target populations ( Al Dandan *et al.*, 2019). The perspectives and beliefs of participants will be gathered using semi-structured focus group interviews via Zoom. The interview guide and all participant documents can be found as extended data ( Houghton *et al.*, 2021).

***Research team roles and prior experience.*** The research team was established through the Health Research Board- Trials Methodology Research Network (HRB-TMRN) and MRC-NIHR Trials Methodology Research Partnership (TMRP) with researchers who share a common interest in qualitative data sharing in trials. Participants will be informed that the research is funded by the HRB- TMRN in collaboration with the MRC-NIHR TMRP and the purpose of the study is clearly stated in the participant information leaflets ( Houghton *et al.*, 2021). All focus groups will be moderated and analysed by CH, LB and MD with assistance from MMC. CH, LB and MD have extensive experience in qualitative methodologies, qualitative evidence synthesis and qualitative data analysis. KG, NR and JW will provide methodological expertise and advise on how to recruit qualitative researchers in trials in the UK. CG will advise on existing guidance for data sharing in clinical trials and implications for qualitative data. ET has expertise in health research transparency and trial methodology and will provide input on all aspects of the project. KMS will provide guidance and support in the scoping activities to identify existing practices in trials. MS will provide advice on how best to access the UK trial community and will assist with the dissemination of the project findings. VB will advise on the development of the focus group interview guide for trial participants and will advise on recruitment for the focus group interview.

***Sampling and recruitment.*** We will use a maximum variation approach to sampling to capture the views and experiences of different stakeholder groups across the UK and Ireland ( Patton *et al.*, 2008). We will conduct four focus groups of 6–8 key stakeholders each, from the UK and Ireland to explore their perspectives of qualitative data sharing in clinical trials. This will equal approximately 32 interviewees. The sample size for this study will follow guidance from Vasileiou *et al.* (2018) regarding ‘data adequacy’, whereby we aim to have sufficient data for meaningful analysis capturing the perspectives of those involved in qualitative research in clinical trials. In the context of this study, “data adequacy” appears more suitable than “data saturation” in the process of decision-making regarding sample sizes, given that the concept of data saturation is often poorly defined within qualitative studies ( Sebele-Mpofu, 2020). Separate focus group interviews will be conducted with members of each stakeholder group: trial managers, clinical trialists, qualitative researchers, members of research funding bodies and trial participants who have been involved in qualitative research.

The aim is to recruit participants who have had experience of qualitative data in clinical trials either as researchers, participants in trials or as funders reviewing grant application for clinical trials. We will recruit participants by contacting the UK Clinical Research Collaboration (UKCRC), Irish Clinical Research Facilities (CRF) and through Health Research Board Trials Methodology Research Network (HRB TMRN) and MRC-NIHR TMRP networks. We will ask them to circulate a recruitment email through their respective mailing lists and social media channels.

Once prospective participants express an interest in engaging with the study, they will be sent the participation information leaflet (see extended data) and will be required to complete an online consent form prior to their focus group (see extended data). Participants will be informed of the interview procedures and the recordings at least one week in advance of the research study. Participants will be provided with contact information for the research assistant (MMC) if they have any questions in advance, and it will be emphasised that consent in the research study is completely voluntary. Informed consent will be obtained prior to any data collection. Participants will also be required to consent to the use of recordings. Participation in focus groups will only commence once informed consent from all participants is received. Due to the minimally invasive nature of this study, as well as the familiarity with participants regarding research ethics and the research process, it is not anticipated that the study will cause any discomfort or distress. It is also not anticipated that the study will causes any discomfort or distress as participants can withdraw at any stage of the focus group. However, if participants do become distressed during the course of the interviews, a distress protocol (see extended data) will be implemented by the interviewers. While participants can withdraw at any point during the focus group, upon focus group completion it will not be possible to withdraw individuals due to the group format of the recording.

**Inclusion criteria:**

•       Aged 18 years or over

•       Any of the following:

•       Trial managers, clinical trialists, and qualitative researchers who have experience of qualitative research in clinical trials.

•       People working with trial funding agencies who have experience of reviewing grant applications for clinical trials with qualitative components.

•       People who have participated in a completed clinical trial where data has been collected using qualitative methods.

**Exclusion criteria:**

•      Individuals under 18 years of age

•      Individuals with impaired capacity to consent independently

•      Individuals who are unable to understand or speak in English

•      Individuals currently still enrolled in a clinical trial

***Data collection.*** Focus groups are a valuable method of data collection in qualitative research ( Tausch & Menold, 2016). In comparison to individual interviews, which aim to explore individual attitudes, beliefs and feelings, focus groups elicit a multiplicity of views and use the process of interactions between participants to generate additional ideas and clarifications ( George, 2013). Focus groups enable researchers to obtain a large amount of information within a short period of time.

This study will employ online methods of data collection as face-to-face contact is not possible due to current coronavirus disease 2019 (COVID-19) restrictions, and virtual focus groups offer the opportunity to bring people together who are not geographically co-located. Focus group interviews will be conducted virtually using a secure Zoom video conferencing account and will be audio-visually recorded. In addition, field notes will be taken during the focus groups as they maintain contextual details and non-verbal expressions for data analysis and interpretation ( Houghton *et al.*, 2013; Tong *et al.*, 2007). Focus groups are expected to be one-off, and approximately one hour in duration. Participants will be made aware that focus group data cannot be withdrawn once the interview is finished but they do not have to answer particular questions if not comfortable doing so. 

We have developed a semi-structured interview guide (see extended data), that explores perspectives of sharing qualitative data, potential benefits and challenges of same, and recommendations for what guidance is needed to support those involved in sharing qualitative data. A semi-structured topic guide consists of open-ended questions and will help the researcher to remain flexible by adapting questions and elaborating on ideas ( Willig, 2013). This method will help generate rich data as it allows participants to build on one another’s statements and comments ( Guest *et al.*, 2013; Tong *et al.*, 2007).

***Data protection.*** As focus group data will be collected through online methods, procedures for data collection and processing will follow the six principles of the European (GDPR), and the Irish Data Protection Act, 2018. For focus groups, participants are asked to comply with confidentiality within the group and will sign consent to this prior to commencing the group discussion. Online focus groups will be conducted via a secure Zoom account. Online focus groups will be moderated by a member of the research team and will be recorded.

When transcription is completed, audio visual recordings will be destroyed and only the anonymised transcripts with the pseudonyms will be retained. In accordance with NUIG Policy, transcripts, field notes and documents will be retained for a minimum of seven years. In accordance with GDPR 2018 and NUIG Personal Data Security Schedule (PDSS), electronic records will be held on the NUI Galway One Drive server accessed through a password protected, encrypted laptop belonging to the lead researcher. All responses will remain confidential and individual names will not be directly linked to individual responses at any time during or after the study. All interviewee responses will be pseudonymised prior to reporting of the results. Pseudonymised findings may be shared with members of the wider project research team to assist with and/or inform data analysis; however, all identifiable information will be removed and individual responses will not be reported. Only members of the research team based in NUIG will have access to the raw data collected, it will not be shared with anyone else, though a professional transcription company will be employed under strict data confidentiality agreements to transcribe the interviews.

***Topic guides.*** The interview topic guide and questions were developed by members of the research team by reviewing existing literature regarding the sharing of qualitative data in clinical trials. The interview questions were based on the principles of developing semi-structured interviews in qualitative research. The interview guides for each stakeholder group are available as extended data.

***Data analysis.*** Data from focus groups will be analysed using thematic analysis ( Braun & Clarke, 2006). Thematic analysis is a flexible method which enables researchers to focus on the data in numerous different ways ( Braun & Clarke, 2006). Thematic analysis will be carried out in line with the six key steps outlined by Braun and Clarke.

**Step one: Familiarisation.** The first step involves the researcher(s) becoming familiar with and engaging with the data by reading and re -reading transcripts.

**Step two: Coding.** This step begins once the researcher is familiar with the data and involves generating initial codes across the data set.

**Step three: Generating themes.** Searching for themes will begin and visualising how different codes may combine to form an overarching theme commences.

**Step four: Reviewing themes.** Step four will involve reviewing potential themes and will require questioning the boundaries of and judging whether there is sufficient data to support each theme.

**Step five: Defining and naming themes**. Clear definitions and names will be established for each theme in this stage.

**Step six: Writing up.** This step involves producing the final report which is achieved by weaving together the themes in a logical and meaningful manner ( Braun & Clarke, 2006).

### Work package 2

***Study design.*** This study will also employ a documentary analysis of current (i.e. past 5 years) consent procedures for qualitative data collected as part of the conduct of clinical trials.

***Data collection.*** We will contact trials managers and individual researchers involved in using qualitative data in trials and by contacting UKCRC, Irish Clinical Research Facilities (CRF), and through HRB TMRN and MRC-NIHR TMRP networks, we aim to explore current consent procedures for qualitative data collected as part of the conduct of trials. We will develop a tailored data extraction form to extract data such as whether specific clauses for data sharing are included in the consent form. CH and MMC will review documents including participant information leaflets and consent forms for the qualitative components in trials. We aim to capture consent procedures for approximately forty qualitative studies in trials.

***Data analysis.*** We will use a tailored extraction form to extract data such as whether specific clauses for data sharing are included in the consent form. We will use content analysis to analyse whether and how consent is being obtained for qualitative data sharing ( Elo & Kyngäs, 2008). Content analysis is a valuable method for analysing qualitative material and seeks to analyse data in view of the meanings someone attributes to them ( Krippendorff, 2018). This will provide baseline information on the prevalence of qualitative data sharing as well as the strategies being employed to do so. We will also analyse the purpose for which sharing of qualitative data is being requested, for example, within a group of trials, or for broader open science purposes.

The findings from both the focus group interviews and the documentary analysis of current consent procedures, will provide an insight into what is happening currently, the challenges and opportunities, and what is needed in terms of best practice guideline development for sharing qualitative data in trials.

***Rigour.*** The research team will agree the coding and theme development from the qualitative phase to ensure the data is represented sufficiently in the developed themes which will minimise researcher bias. The analysis will be managed within QSR Nvivo version 12 to provide a transparent audit trail of the decisions made through the analysis ( Houghton *et al.*, 2013). We will utilise the queries tools in Nvivo to enable us to ask ‘questions’ of the analysis ( Houghton *et al.*, 2013). A codebook will be created within QSR NVivo to exhibit the reliability and credibility of our findings.

***Ethics approval.*** We have received ethical approval from NUI Galway Research Ethics Committee (REF:2021.01.009). 

It is important to note that the raw transcript materials generated during the study will be confidential. Only members of the research team will have access to the raw transcript materials. 

***Dissemination.*** The findings of both work packages will be presented to the TMRP Trial Conduct and Health Informatics working groups in a format of their preference, for example as part of the MRC-NIHR TMRP and the HRB-TMRN webinar series. We will also use the study findings to inform a consensus building workshop, following project completion, to identify model data sharing documents to act as an interim source of good practice guidance pending development of comprehensive guidance, and to make these available for the trials community via MRC-NIHR TMRP Trial Conduct Qualitative Research in trials (QRiT) target group. The consensus process will involve forming an advisory panel from the QRiT target group, thus providing an opportunity for members of this group to help drive this agenda and contribute their expertise in this area. This will also highlight the MRC-NIHR, TMRP/HRB-TMRN work in this area, facilitating networking for subsequent activity. We will use the findings to inform further grant applications to develop more cohesive best practice recommendations guidance on sharing qualitative data collected in clinical trials. We will develop a plain language summary and disseminate the findings through social media and websites such as MRC-NIHR TMRP, HRB–TMRN and Qualitative research in Trials Centre (QUESTS). 

***Study Status.*** Recruitment for this study commenced in March 2021.

## Conclusion

This study will provide insight into the existing practice of sharing of qualitative data in clinical trials and the current issues and opportunities, to help shape future research and development of guidance to encourage maximum learning to be gained from this valuable data.

## Data availability

### Underlying data

No data is associated with this article.

### Extended data

Open Science Framework: Qualitative data sharing practices in clinical trials in the UK and Ireland: Towards the production of good practice guidance. https://doi.org/10.17605/OSF.IO/BKC8D. ( Houghton *et al.*, 2021).

This study contains the following extended data:

•      Appendix 1: Recruitment emails

•      Appendix 2: Participant Information leaflets

•      Appendix 3: Informed consent form

•      Appendix 4: Distress Protocol

•      Appendix 5: Focus group Interview guide

Data are available under the terms of the https://creativecommons.org/licenses/by/4.0/legalcode. (CC-BY 4.0)
