# Supplementary material for: A study protocol of qualitative data sharing practices in clinical trials in the UK and Ireland: towards the production of good practice guidance
Source: HRB Open Res. 2021 Jun 22;4:47. Originally published 2021 May 10. [Version 2] doi: 10.12688/hrbopenres.13269.2 (PMC8167499; doi:10.12688/hrbopenres.13269.2)
Supplement: Peer review history

# Peer review: Reviewer response for version 1

**DOI:** 10.21956/hrbopenres.14442.r29417

**Published:** 2021-05-28

**Authors:** Spencer Phillips Hey

**Affiliations:** 1 Center for Bioethics, Harvard Medical School, Boston, MA, USA

Data sharing is an increasingly important issue for trials, particularly as the volume of trials -- and therefore the volume of trial data potentially available for secondary use -- continues to grow at such an incredible pace. While I have encountered several studies examining the logistical and ethical challenges associated with sharing patient-level, quantitative data, this protocol is the first I’ve seen describing a plan to explore issues around sharing of qualitative data. I thus found this protocol paper to be a welcome development, and the proposed research strikes me as a valuable contribution aimed at addressing an important gap in knowledge.

 However, similar to the previous reviewer, I do think there are some sections in this paper that need greater discussion and clarification.

 Foremost among these (for me) is the subsection on Rigour and the thematic analysis: I think much more should be said about how this project will control for bias – and in particular, the possibility that the themes identified will reflect (or be strongly biased towards) the interest, concerns, or assumptions of the research team. For example, I note that the focus group interview guide (Appendix 5) includes prompts about potential benefits (question 4), challenges (question 5), and recommendations (question 6) for sharing qualitative data, but it does not include a question about potential harms. Perhaps “challenges” is intended to encompass the potential for harm, but it is not clear to me that it does and I would not interpret the word in that way. I’d thus strongly suggest adding a dedicated prompt about potential harms (e.g., to privacy or dignity) and being clear to distinguish this from the prompt about logistical/technical/social “challenges”.

 The one sentence in the protocol on this potential for bias currently reads: “The research team will agree [to?] the coding and theme development from the qualitative phase to ensure the data is represented sufficiently in the developed themes which will minimise researcher bias.” I suspect there may be a grammatical error here, which I’ve tried to correct with my bracketed addition. But even the corrected sentence doesn’t really tell me much about what will be done to avoid bias. In fact, since my concern is that the results may be biased toward the research team’s interest, this sentence would only seem to confirm that this bias is "baked in" to the study plan.

 Following on this, I worry also about the potential for bias arising from the sample size and sampling strategy. 32 interviewees strikes me as a rather small number of participants, particularly if this 32 will be a heterogeneous mix of five different stakeholders (trial managers, clinical trialists, qualitative researchers, members of research funding bodies, and trial participants who have been involved in qualitative research). I am not an expert in qualitative research, so this concern may be misplaced, but even looking up the methodological references provided in the protocol suggests that 6 interviews per group is on the low side to achieve “data adequacy” (e.g., Vasileiou *et al*. 2018). (Although I confess I found that Vasileiou paper somewhat difficult to decipher as a methodological guidance document, since it is itself, a thematic analysis of qualitative research methods...)

 Similarly for the recruitment strategy: Leveraging research networks in the UK and Ireland makes complete sense from a pragmatic perspective. However, this strategy seems likely to miss individuals who may have had negative experiences with qualitative data sharing (e.g., because they may have specifically tried to disconnect themselves from established research institutions), and I think it would critical to try and capture at least some of this perspective.

 But to be clear: None of this makes me think that this paper should not be approved. I believe the overarching questions are important and the gap in knowledge is real. I also believe the research team has likely thought much about all the issues I've raised. I think the manuscript could thus be improved  by sharing more of that thinking, e.g., expanding the discussion of the potential for bias and steps taken to ensure (as much as possible) that critical perspectives (i.e., valid arguments from stakeholders who think that qualitative data should not be shared) are represented.

Is the study design appropriate for the research question?

Yes

Is the rationale for, and objectives of, the study clearly described?

Yes

Are sufficient details of the methods provided to allow replication by others?

Yes

Are the datasets clearly presented in a useable and accessible format?

Yes

Reviewer Expertise:

Research ethics, philosophy of science, data science and communication

I confirm that I have read this submission and believe that I have an appropriate level of expertise to confirm that it is of an acceptable scientific standard.

# Peer review: Reviewer response for version 1

**DOI:** 10.21956/hrbopenres.14442.r29419

**Published:** 2021-05-24

**Authors:** Nicola Howe

**Affiliations:** 1 Newcastle Clinical Trials Unit, Newcastle University, Newcastle upon Tyne, UK

Overall I found this protocol paper interesting and well thought out. The rationale and methods are clearly stated. The research (and guidance) gap around sharing of qualitative trial data and the contribution that this research will make, and how the results will be disseminated are all available in this protocol. However, there are several areas of clarification that would be useful for readers that the authors may like to expand upon in order that this research could be truly reproducible. I have listed these below for the authors’ consideration.

**Work package 1:**

 It might be useful to expand the point about the development of the interview topic guide. The authors mention that they reviewed existing literature regarding sharing of qualitative data in clinical trials, but did they model any of their questions on those in the existing literature, with modifications? If so, which existing literature was used? The authors could refer briefly to the papers here.

 I understand the exclusion criteria, but it might be useful for the authors to state explicitly why participants should no longer be taking part in a clinical trial.

 Can the authors expand upon/confirm that the coding in NVivo was data driven or theory driven? The authors don’t mention using a framework to code, but can they confirm that they did not and that the coding was instinctive?

 It might be useful for the authors to state which researcher will conduct the coding, or whether more than one researcher will do this. The authors do make clear that there will be team consensus on codes and themes afterwards to reduce bias but will there be consensus during coding?

**Work package 2:**

 Could the tailored extraction form be provided in extended data?

Is the study design appropriate for the research question?

Yes

Is the rationale for, and objectives of, the study clearly described?

Yes

Are sufficient details of the methods provided to allow replication by others?

Partly

Are the datasets clearly presented in a useable and accessible format?

Not applicable

Reviewer Expertise:

clinical trials, data sharing, data management, participant's attitudes towards data sharing,

I confirm that I have read this submission and believe that I have an appropriate level of expertise to confirm that it is of an acceptable scientific standard.
